# Supplementary material for: Emergent discrete space-time crystal of Majorana-like quasiparticles in chiral liquid crystals
Source: Nat Commun. 2026 Mar 24;17:4376. doi: 10.1038/s41467-026-70880-8 (PMC13179321; doi:10.1038/s41467-026-70880-8)
Supplement: Supplementary file 1 — Supplementary Information [file 41467_2026_70880_MOESM1_ESM.pdf]

Supplementary Information

Emergent discrete space-time crystal of Majorana-like quasiparticles in chiral liquid crystals

Hanqing Zhao[1,2], Rui Zhang[2,3,4] and Ivan I. Smalyukh[1,2,5,6*]

[1]*Department of Physics, University of Colorado, Boulder, CO 80309, USA*

[2]*International Institute for Sustainability with Knotted Chiral Meta Matter (WPI-SKCM²), Hiroshima University, Higashi Hiroshima, Hiroshima 739-8526, Japan*

[3]*Department of Physics, The Hong Kong University of Science and Technology, Clear Water Bay, Kowloon, Hong Kong 99999, People's Republic of China*

[4]*State Key Laboratory of Displays and Opto-Electronics, The Hong Kong University of Science and Technology, Clear Water Bay, Kowloon, Hong Kong 99999, People's Republic of China*

[5]*Materials Science and Engineering Program, University of Colorado, Boulder, CO 80309, USA*

[6]*Renewable and Sustainable Energy Institute, National Renewable Energy Laboratory and University of Colorado, Boulder, CO 80309, USA*

* *Correspondence to: ivan.smalyukh@colorado.edu*

Supplementary Figures

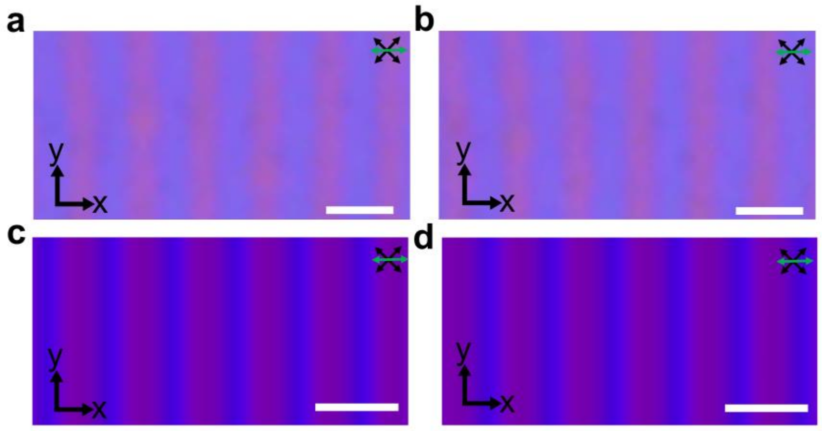

Supplementary Fig. S1| POM images of quasi-static initial director structures. a,b, Experimental POM images from two neighbouring driving periods when the voltage is close to

zero. **c**,**d**, Numerically simulated quasi-static initial **n**(**r**) corresponding to Fig. 3a,b. Scale bars indicate 10 μm.

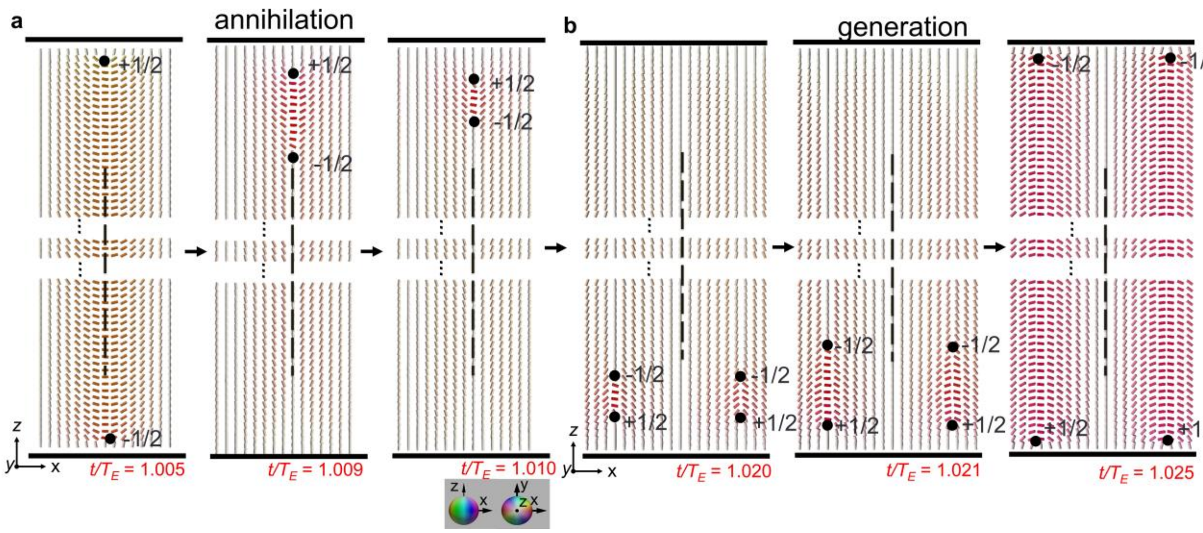

Supplementary Fig. S2| Details of the dynamics of topological quasiparticles. a, Snapshots of the director field showing the annihilations of quasiparticles. **b,** Snapshots of the director field showing the generation of quasiparticles at locations shifted by half a spatial period from the ones in the previous electric driving period. Black solid circles indicate the disclination cores; black dashed lines indicate relative positions for reference.

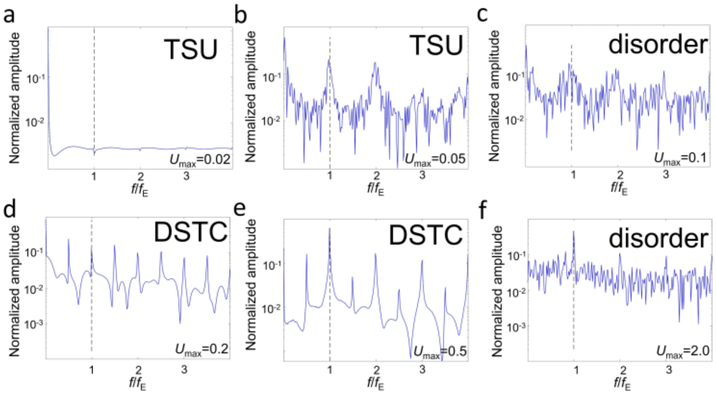

Supplementary Fig. S3| Numerically simulated results for different voltage amplitudes. a-f, FFT analysis of the time-dependent director field for low to high voltage amplitudes, showing the time-symmetry-unbroken (the temporal periodicity is the same as the external drive), period-doubling, and disordered (a disordered response to the external drive) behaviours. The frequency f is normalized by the external drive frequency f_E.

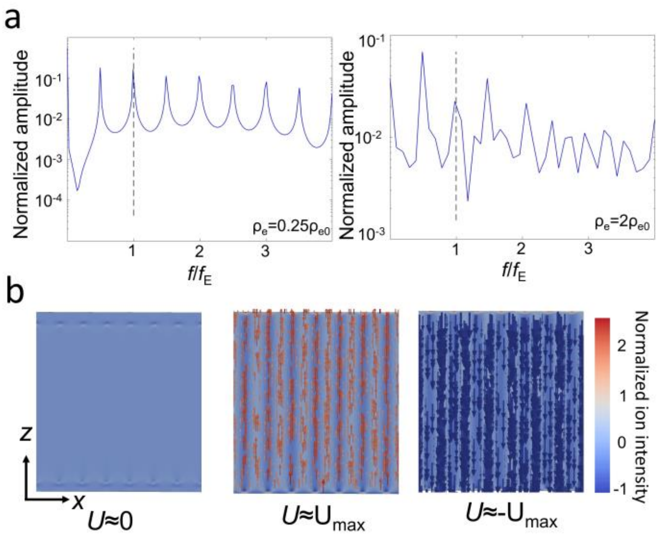

Supplementary Fig. S4| The effects of ion concentration and ionic current. a, Fast Fourier transform analysis of the time-dependent director field for different ion concentrations ρ_e in simulation, where ρ_{e0} is set as the unit. The period-doubling phenomenon does not occur if the ion concentration is below or above the threshold, consistent with the experiments. **b,** Ionic current is represented by arrows at different voltage amplitudes within one temporal period; the background is coloured according to the normalized ion intensity (right-side colour scheme inset).

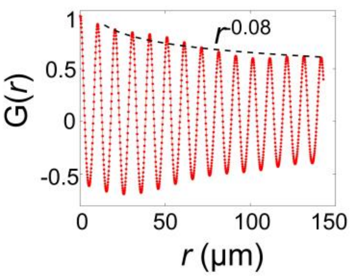

Supplementary Fig. S5| Space correlation function of 1+1D DSTCs.

Correlation function versus distance *r*, where *r* is along the *x* direction in Fig. 1d. The fitting of the curve (black dashed line) may indicate a power-law $r^{-0.08}$ decay.

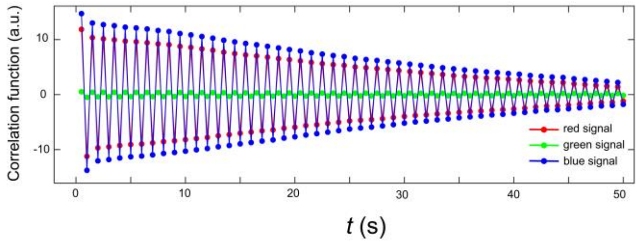

Supplementary Fig. S6| Time-dependent correlation functions for RGB signals.

Correlation function versus time for the red, green, and blue signals, respectively. The time interval $T_E = 0.5$s.

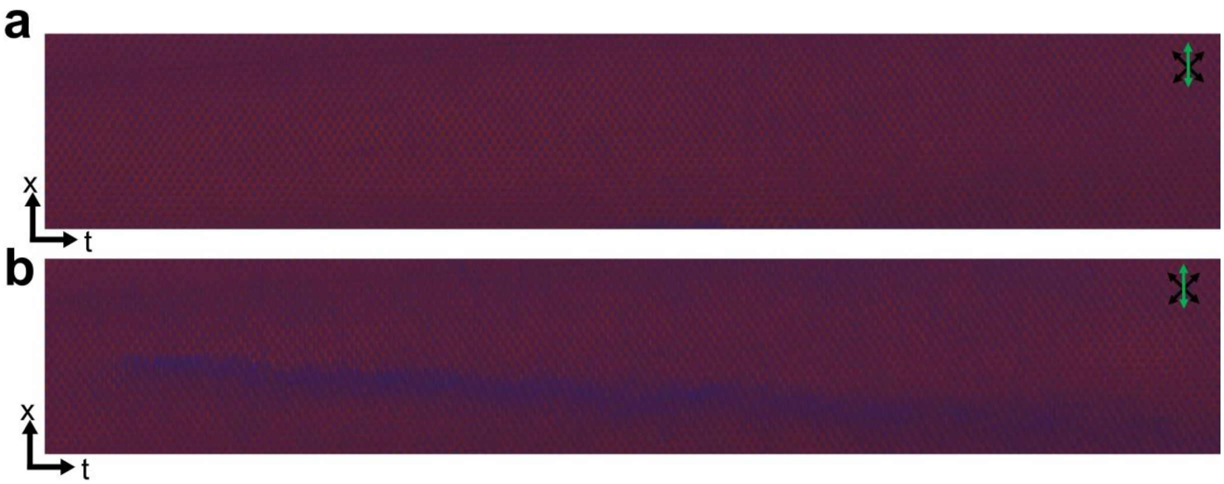

Supplementary Fig. S7| Space-time images of a 1+1D DSTC. a, Space-time image of a 1+1D DSTC over 250 external drives. **b,** Space-time image of a 1+1D DSTC with impurity regions. The time interval $T_E = 0.5$s.

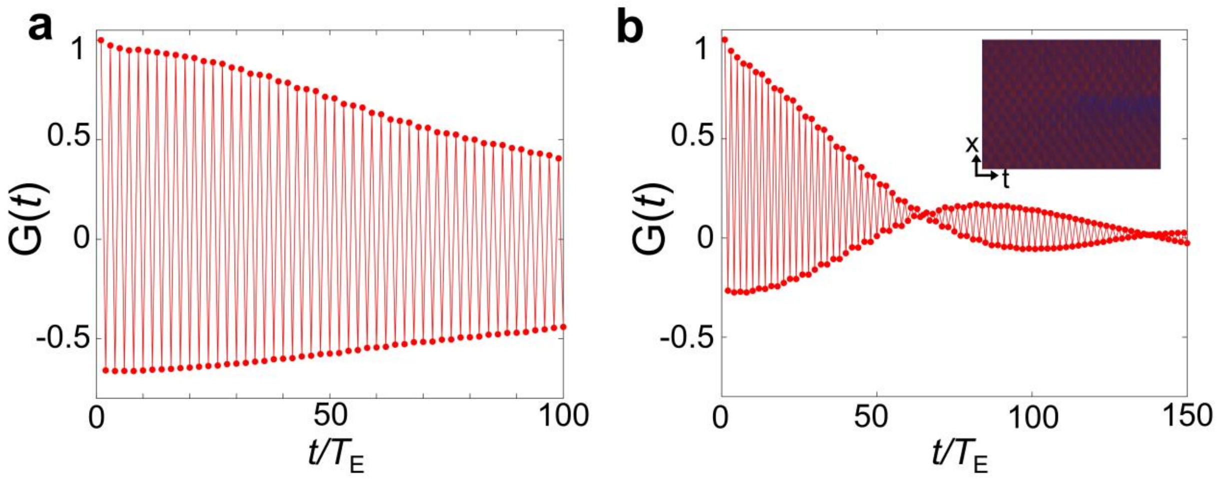

Supplementary Fig. S8| Time-dependent correlation functions averaged over different driving durations. a, Correlation function versus time of a 1+1D DSTC, averaged over 250 drives, corresponding to Supplementary Fig. S7a. **b,** Correlation function versus time of a 1+1D DSTC, averaged over 1500 drives, including impurity regions shown in Supplementary Fig. S7b. The impurity region (inset in b) tends to destroy the correlation in the long ranges; therefore, achieving extended correlations requires enhancing system controls that preclude issues like thermally- or vibrations-induced drift.

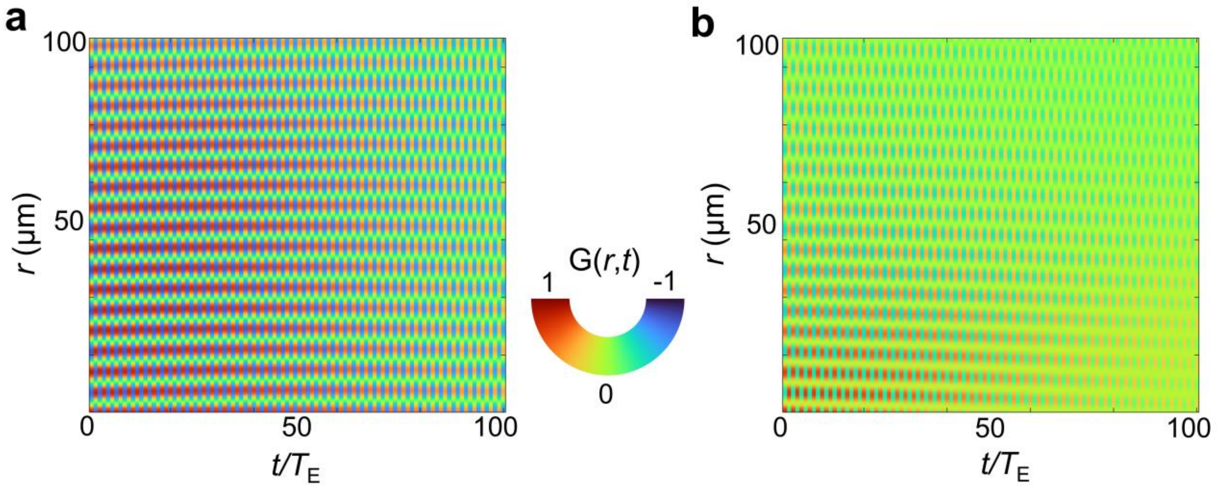

Supplementary Fig. S9| Space-time correlation functions over different driving durations.

a, Correlation function as a function of space *r* and time *t* for a 1+1D DSTC, averaged over 250 drives, corresponding to Supplementary Fig. S7a. **b,** Correlation function as a function of space *r* and time *t* for a 1+1D DSTC, averaged over 1,500 drives, including the impurity regions shown in Supplementary Fig. S7b. The space-time correlation function G(*r*,*t*) is coloured according to the scheme shown in the middle inset.

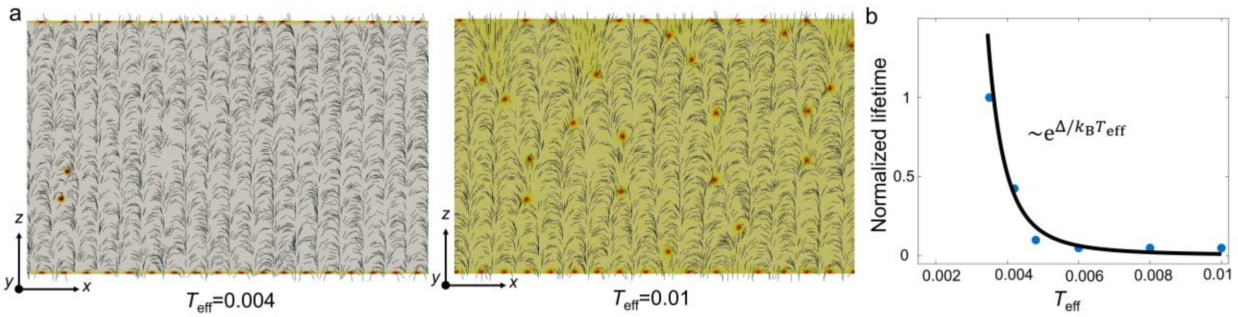

Supplementary Fig. S10| Numerically simulated lifetime of the 1+1D DSTC. **a,** Snapshots of **n**(**r**) at different effective temperature T_{eff} of the thermal perturbation, the background is coloured by the scalar order parameter *S*. The order of the system decreases and more defects (dark red regions) emerge as the T_{eff} increases. **b**, Normalized lifetime (correlation time) versus T_{eff}. The lifetime can be fitted by $\sim e^{\Delta/k_{\mathrm{B}}T_{\mathrm{eff}}}$, where Δ is the quasienergy activation barrier and k_{B} is the Boltzmann constant, indicating a potentially (thermally) activated time crystal (Ref. [9]). The lifetime τ_0 is normalized by the maximum simulation time when $T_{\mathrm{eff}} < 0.0035$.

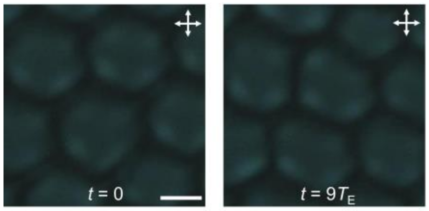

Supplementary Fig. S11| POM images of a potential candidate of fractional DSTC. POM snapshots captured with an interval of 9T_E, showing that the system does not have a strict 1/3-subharmonic response, since the images are not identical when shifted by an integer multiple of 3T_E. Transmitting axes of the polarizer and analyser are marked by white double arrows. Scale bars indicate 10 μm.
