# Supplementary material for: Emergent discrete space-time crystal of Majorana-like quasiparticles in chiral liquid crystals
Source: Nat Commun. 2026 Mar 24;17:4376. doi: 10.1038/s41467-026-70880-8 (PMC13179321; doi:10.1038/s41467-026-70880-8)
Supplement: Supplementary file 2 — Description of Additional Supplementary Files [file 41467_2026_70880_MOESM2_ESM.docx]

File Name: Supplementary Movie 1

Description: Movies showing the 1+1D and 2+1D DSTCs. The POM movie for 1+1D DSTC (top left) is obtained for a cell gap *d* = 5 μm, the POM movies for configuration 1 (top right) and configuration 2 (bottom left) 2+1D DSTCs are obtained for cell gaps *d* = 10 μm, and the POM movie for configuration 3 (bottom right) 2+1D DSTC is obtained for a cell gap *d* = 15 μm. The external drive periodicity *T*_E_ = 0.5s. The elapsed time (in units of *T*_E_) and scale bar are marked on the movie frames. The transmitting axes of the polarizer and analyser are marked by black double arrows. The slow axis of the retardation plate is marked by a green double arrow.

File Name: Supplementary Movie 2

Description: Movies showing director field and scalar order parameter intensity when *U* is close to zero. Numerically simulated director field (left) based on the Landau-de Gennes free energy functional, the background is coloured by the scalar order parameter *S* where the colouring scheme is the same as Fig. 4. The movies in the right sides are the zoomed-in region marked on the left. The corresponding voltage is marked on the movie frames.

File Name: Supplementary Movie 3

Description: Phases of DSTCs. POM movies showing the time-symmetry-unbroken phase (left), disordered phase (middle) and co-existence phase (right), respectively. The POM movies are obtained for *d* = 5 μm. The elapsed time and scale bar are marked on the movie frames. The transmitting axes of the polarizer and analyser are marked by black double arrows. The slow axis of the retardation plate is marked by a green double arrow.

File Name: Supplementary Movie 4

Description: Movies showing DSTCs under different conditions. The POM movies are obtained for *d* = 5 μm, *T*_E_ = 0.35 s and *U*_max_ = 90 V (top left), *d* = 5 μm, *T*_E_ = 0.6 s and *U*_max_ = 90 V (top middle), *d* = 5 μm, *T*_E_ = 1.0 s and *U*_max_ = 90 V (top right), *d* = 10 μm, *T*_E_ = 0.3 s and *U*_max_ = 50 V (bottom left), *d* = 10 μm, *T*_E_ = 0.6 s and *U*_max_ = 50 V (bottom middle) and *d* = 10 μm, *T*_E_ = 0.9 s and *U*_max_ = 90 V (bottom right), respectively. The elapsed time and scale bar are marked on the movie frames. The transmitting axes of the polarizer and analyser are marked by black double arrows. The slow axis of the retardation plate is marked by a green double arrow.

File Name: Supplementary Movie 5

Description: Formation of the 2+1D DSTC. POM movie showing dynamics of the 2+1D DSTC “boil out” from a disordered state. The POM movie is obtained for a cell gap *d* = 10 μm, and the external drive periodicity *T*_E_ = 0.5s. The elapsed time and scale bar are marked on the movie frames. The transmitting axes of the polarizer and analyser are marked by black double arrows.The slow axis of the retardation plate is marked by a green double arrow.

File Name: Supplementary Movie 6

Description: Rigidity of the 2+1D DSTC against temporal perturbations. POM movie showing the 2+1D DSTC against temporal perturbations $\Delta$*T*_E_ randomly distributed within [-0.2$\bar{T}_{E}$,+0.2$\bar{T}_{E}$] (left) and [-0.4$\bar{T}_{E}$,+0.4$\bar{T}_{E}$] (right), where $\bar{T}_{E}$ = 0.5s. The POM movies are obtained for cell gaps *d* = 10 μm. The elapsed time and scale bar are marked on the movie frames. The transmitting axes of the polarizer and analyser are marked by black double arrows. The slow axis of the retardation plate is marked by a green double arrow.

File Name: Supplementary Movie 7

Description: Emergence and disappearance of a defect region in a 2+1D DSTC. To clearly show the dynamics, we capture the snapshots with temporal interval *T*_E_, and then compile them into a movie. The POM movie is obtained for a cell gap *d* = 10 μm, and the external drive periodicity *T*_E_ = 0.5s. The scale bar is marked on the movie frames. The transmitting axes of the polarizer and analyser are marked by black double arrows. The slow axis of the retardation plate is marked by a green double arrow.

File Name: Supplementary Movie 8

Description: Healing of 1+1D DSTC after generating a defect by a laser tweezer. The POM movie is obtained for a cell gap *d* = 5 μm, and the external drive periodicity *T*_E_ = 0.5s. The elapsed time and scale bar are marked on the movie frames. The transmitting axes of the polarizer and analyser are marked by black double arrows. The slow axis of the retardation plate is marked by a green double arrow.

File Name: Supplementary Movie 9

Description: Movie showing the 1+1D DSTC over long driving time periods. The POM movie is obtained for a cell gap *d* = 5 μm, and the external drive periodicity *T*_E_ = 0.5s. The elapsed time and scale bar are marked on the movie frames. The transmitting axes of the polarizer and analyser are marked by black double arrows. The slow axis of the retardation plate is marked by a green double arrow.

File Name: Supplementary Movie 10

Description: An ordered quasi-hexagonal lattice under external Floquet drives. The external drive periodicity *T*_E_ = 0.5s. The elapsed time (in units of *T*_E_) and scale bar are marked on the movie frames. The transmitting axes of the polarizer and analyser are marked by white double arrows in the left and black double arrows in the right. The slow axis of the retardation plate is marked by a green double arrow.
